# Running away experience and psychoactive substance use among adolescents in Taiwan: multi-city street outreach survey

**DOI:** 10.1186/1471-2458-10-29

**Published:** 2010-01-20

**Authors:** Shi-Heng Wang, Wen-Chun Chen, Chih-Yin Lew-Ting, Chuan-Yu Chen, Wei J Chen

**Affiliations:** 1 Institute of Epidemiology, College of Public Health, National Taiwan University, 17 Xuzhou Road, Taipei 100, Taiwan; 2 Department of Public Health, College of Public Health, National Taiwan University, 17 Xuzhou Road, Taipei 100, Taiwan; 3 Institute of Health Policy and Management, College of Public Health, National Taiwan University, 17 Xuzhou Road, Taipei 100, Taiwan; 4 Division of Mental Health and Addiction Medicine Research, National Health Research Institutes, 35 Keyan Road, Zhunan, Miaoli County 350, Taiwan; 5 Department of Psychiatry, College of Medicine and National Taiwan University Hospital, National Taiwan University, 7 Chung-Shan South Road, Taipei 100, Taiwan

## Abstract

**Background:**

This study aimed to examine: 1) the relationship between being a runaway and the time since the first absconding event and adolescent substance use; 2) whether different kinds of psychoactive substances have a different temporal relationship to the first absconding event; and 3) whether the various reasons for the first absconding event are associated with different risks of substance use.

**Methods:**

Participants were drawn from the 2004-2006 nationwide outreach programs across 26 cities/towns in Taiwan. A total of 17,133 participants, age 12-18 years, who completed an anonymous questionnaire on their experience of running away and substances use and who were now living with their families, were included in the analysis.

**Results:**

The lifetime risk of tobacco, alcohol, betel nut, and illegal drug/inhalant use increased steadily from adolescents who had experienced a trial runaway episode (one time lasting ≤ 1 day), to those with extended runaway experience (≥ 2 times or lasting > 1 day), when compared to those who had never ran away. Adolescents who had their first running away experience > 6 months previously had a greater risk of betel nut or illegal drug/inhalant use over the past 6-months than those with a similar experience within the last 6 months. Both alcohol and tobacco use were most frequently initiated before the first running away, whereas both betel nut and illegal drug/inhalant use were most frequently initiated after this event. When adolescents who were fleeing an unsatisfactory home life were compared to those who ran away for excitement, the risk of alcohol use was similar but the former tended to have a higher risk of tobacco, betel nut, and illegal drug/inhalant use.

**Conclusions:**

More significant running away and a longer time since the first absconding experience were associated with more advanced substance involvement among adolescents now living in a family setting. Once adolescents had left home, they developed additional psychoactive substance problems, regardless of their reasons for running away. These findings have implications for caregivers, teachers, and healthcare workers when trying to prevent and/or intervening in adolescent substance use.

## Background

Runaway or homeless adolescents are a high risk group for using a variety of psychoactive substances [1,2]. The greater availability of illegal psychoactive substances and the tremendous stress faced on the streets increase the runaway youth's risk of engaging in drug-related activities once they leave home [3-5]. Given the complicity and heterogeneity that exist in interrelationships between runaways and substance use, researchers have come to accept the notion that the behaviour of absconding from home *per se *should be examined as a continuum, ranging from leaving home for a few hours to living on the streets for an extended period of time [6]. For instance, among those who experienced short-term running away, only a small minority of them go on to become homeless or street adolescents [7].

Adolescents with experience as runaways are less likely to be sampled in school-based or household-based surveys [8]. Therefore, most studies to date on psychoactive substance use among runaway adolescents have recruited their participants either from shelters [9-13] or from the streets [14-17], which represent the tip of the iceberg of this population. Less explored, but important from the viewpoint of early detection and intervention, is the risk of psychoactive substance use among adolescents who are still living with their families, but have run away from home at some point. A household survey in the USA indicated that adolescents with overnight absconding within the past 6 months have a higher rate of psychoactive substance use than those without similar experience [18]. However, that study did not distinguish whether the absconding event was the first time or not, which might have a differential effect on substance use. Furthermore, little is known about whether adolescents with experience of a brief running away episode have a high prevalence of substance use, and whether drug involvement differs with the time that has elapsed since the first episode. Previous research has indicated that the reasons for running away may be linked with a varying risk of drug use among adolescents who are staying in shelters or on the streets [19], yet whether such a phenomenon exists among adolescents with brief absconding experiences remains to be investigated.

With few exceptions, which are limited to qualitative interviews with adolescents staying in shelters or juvenile correction centres, runaway adolescents are rarely the topic of explicit attention in the research concerning youth health and wellbeing in Taiwan [20]. Such ignorance of a high-risk group may hinder intervention and prevention programs that target substance use; these problems have been characterized by several social transformations over recent years. One transformation involves an increase in alcohol and tobacco availability to adolescents and a decline in the initiation age for alcohol and betel nut use as a consequence. This is, in part, associated with the trade liberalization and lower custom tariffs on imported tobacco and alcohol beverages since 1987 [21-24]. Another transformation involves the upsurge in availability and diversity of illegal drugs, particularly methamphetamine, ecstasy, marijuana, ketamine, and heroin [25-28].

To address the gaps in the literature, we have turned to adolescent sample recruited from a nationwide multi-city street outreach that covered three consecutive years. The aims of this study are: 1) to investigate associations linking running away, in terms of severity and time since the first absconding episode, with substance use involvement among adolescents who were living with their families at the time of the survey; 2) to examine whether the temporal sequence linking substance use and the first absconding episode may be different across the type of substance used, including alcohol, tobacco, betel nut, and illegal drugs/inhalants; and 3) to examine whether the connection between running away and substance use varies with the reason for absconding.

## Methods

### Study population

The subjects of this study were participants in the outreach arm of the 2004-2006 National Survey of Illegal Drug use among Adolescents (NSIDA) in Taiwan. In an attempt to recruit adolescents from major cities and towns nationwide, the NSIDA divided Taiwan into seven main geographic clusters according to their geographic characteristics, urbanicity, and population composition; several major cities and towns within each geographic cluster were then selected in which the outreach survey was implemented. A total of 26 cities and towns were selected. Detailed information about the study design, sampling, and recruitment procedures has been reported elsewhere [26,29]. The study was reviewed and approved by the Institutional Review Board of the College of Public Health, National Taiwan University.

Subject recruitment was carried out in selected natural settings frequented by teenagers in Taiwan including fast food restaurants, pool halls, cybercafés, and downtown areas. Each outreach team was composed of two or three trained research assistants who carried out the initial approach to the subject, briefly explaining the outreach project, collecting data, and recording key information if the adolescents refused to participate. A quality control process was set up for the fieldwork by means of random inspection. Each assistant was asked to fill out via the Internet a pre-field form before the recruitment day and an on-field form after each day's recruitment. The pre-field form detailed information on expected recruitment time and site, while the on-field form detailed information on practical recruitment time and site, the number of participants, and the number of rejecters. Random inspections of the fieldwork at the sites indicated on the pre-field form were conducted by local supervisors. In order to recruit adolescents with diverse backgrounds and to avoid sampling bias resulting from specific settings or periods, no more than 15 respondents per setting were sampled by one assistant on any given day [26,29]. The recruitment period was between 4:00 pm and 10:00 pm each weekday for the first year in 2004. To capture more adolescents with unstable school attendance, the recruitment time was extended to 9:00 am to 10:00 pm each weekday in 2005 and 2006. After assuring each participant that the survey was anonymous and confidential, an assistant would accompany the adolescent to a more private place to sign an informed consent with a first name or nickname and then fill in the questionnaire. Participants received stationery (less than $1 US) as an incentive once they completed the assessment.

### Study sample

A total of 6014 participants for 2004, 6516 for 2005, and 6405 for 2006 were recruited and completed the questionnaire. Overall, the response rate for this outreach survey was 75.7% in 2004, 70.9% in 2005, and 57.9% in 2006. Male adolescents were more likely to refuse participation compared to their female counterparts across the three years (26.1% vs. 22.7% in 2004, 29.9% vs. 28.3% in 2005, and 42.3% vs. 41.9% in 2006, respectively).After excluding 120 participants who were under 12 years old, 368 who were older than 18 years, 1 with their age missing, 1 with their runaway status missing, and 25 who report the use of a fake drug, the numbers of subjects included in this study for each year were 5886 in 2004, 6264 in 2005, and 6270 in 2006 (total N = 18420). Furthermore, in the context of this study, the sample was then restricted to adolescents living with their families at the time of survey (total N = 17133).

### Measures

The data were collected with a self-administered paper-and-pencil questionnaire that inquires information on personal background (gender, age, attending school or not, single parent family or not, living with family or not, allowance, and employment experience), on development-related behaviours (runaway, truancy, and sexual experience), and on substance use experiences. Adolescents' weekly monetary allowance was adopted as a proxy measure for their family socioeconomic status [30]. Middle or high school students in Taiwan may move away from home in order to attend schools beyond commuting distance from home, living in a school dormitory or rental housing; such persons, who were not living with their family at the time of survey, might be under low levels of parental supervision and these individuals were deleted from the subsequent analyses.

For development-related behaviours, such as running away, truancy, and sexual experience, participants were first asked whether they ever had the experience, and more detailed questions were then asked about the endorsed experience. In assessing the participants' experience of running away, participants were asked whether they had ever run away from home; if they answered yes, further questions were asked about initiation age, frequency, longest time away from home, their recent experiences of running away over the last six months, and the reason for the first running away (the last one only in the 2006 survey). The measures on substance use covered lifetime experiences of tobacco, alcohol, betel nut, and nine kinds of illegal drugs or inhalants (ecstasy, ketamine, marijuana, angel dust, gamma hydroxybutyrate [GHB], methamphetamine, flunitrazepam [so-called FM2], heroin, and glue). Although the selling of tobacco, alcoholic beverages, or betel nut to individuals under the age of 18 is prohibited by law in Taiwan, the enforcement of this regulation by and large varies among shops. Betel nut (or areca nut) is a mild central nervous system stimulant widely used in Asia. Its active principle is the alkaloid arecoline, which stimulates both the parasympathetic and sympathetic nervous systems in a dose-dependent manner [31]. In addition, a "bogus" drug was added to test the validity of self-reported substance use. For those who had ever used each of the substance, more detailed information was asked about age and circumstance of first use, the motive for first use, the average frequency of consumption, the cumulative frequency, and the date of last use.

We classified experience as a runaway in two different ways, namely by severity and by time-since-the first absconding episode. For the classification by severity, adolescents' running away was grouped into a trial running away (only 1 time, lasting 1 day or less, but had not run away in the past 6 months) vs. extended running away (at least 2 times or having the longest runaway episode more than 1 day). For the classification of the time since the first running away, the data were split into within the past 6 months vs. more than 6 months ago. Among those who had run away before (N = 1626), the severity was not associated with how recent was the first absconding episode, with 44 out of 485 (9.1%) having the trial absconding being initiated within the past 6 months vs. 132 out of 1141 (11.6%) having extended absconding being initiated within the past 6 months (odds ratio [OR] = 0.76, *p *= 0.14). In the subsequent analysis by severity, we excluded 44 adolescents who had run away only once, lasting 1 day or less, within past 6 months, because they might not continue to try running away, based on the limited time period between their first running away and the assessment.

Additionally, we classified the reason for the first running away using Zide and Cherry's classification [19]. Since our sample was limited to adolescents who still lived with their families at the time of the survey, none of them fit the categories of "thrown out" or "forsaken." Thus, the reason for the first running away was classified as 'running from an unsatisfactory home life' (e.g., in conflict with or been physically abused by parents), as 'running for excitement,' or as both.

### Data analysis

Since our sample was non-probabilistic, unweighted analysis was adopted. Nevertheless, adolescents living in the same region would presumably be more similar to each other than those living in other regions; thus, we treated each region as a cluster unit to take into account any within region interdependence when estimating standard errors.

Chi-square tests were first performed to examine whether the distribution of personal characteristics and development related behaviours differed in terms of absconding experience. Next, the relationships between running away and substance use were examined in two different ways: 1) the severity of running away vs. *lifetime *substance use (as the dependent variable); and 2) the recentness of the first running away vs. *recent *(past 6 months) substance use (as the dependent variable). *Lifetime *substance use refers to ever having used a substance and *recent *substance use refers to ever having used a substance in the past 6 months. Since the severity of running away was not associated with the recentness of the first running away, we did not adjust for potential confounding between the two by putting them in the same model. To further explore the temporal relationship between the initiation of running away and substance use, subjects were grouped on the basis of which experience occurred first among those who had both absconded and undertaken substance use. Compared to the age for the first absconding event, the proportions of those with a younger or older age at onset of substance use were tested using the chi-square test against the expected proportions of 50% under the null hypothesis of no ordering between the two. In addition, we explored the relationship between the reason of the first running away and substance use. Multivariable logistic regression analysis was used, taking region clustering design effects along with survey year and other potential confounders into account, and was applied to estimate the strength of associations between running away and substance use; this was carried out using the procedure Proc Surveylogistic in the SAS software. All the analyses were conducted using SAS version 9.1 (SAS Institute Inc., Cary, NC). A *p *value of less than 0.05 was considered statistically significant.

## Results

### Sample characteristics

The distributions of sociodemographic and behavioural characteristics across different experiences as a runaway are displayed in Table 1. Compared to adolescents who never ran away, those who had absconded, regardless of the type (trial or extended), showed a higher prevalence of several sociodemographic and behavioural characteristics, including male gender, older age, coming from a single parent family, not attending school, having a job, having a high weekly monetary allowance, having experience of truancy, and having sexual experience. Furthermore, there was an increasing trend in the prevalence of these characteristics that paralleled the severity of running away. The prevalence of truancy increased from 26.1% for those who had never absconded to 62.8% for those with a trial running away, and 80.0% for those with an extended running away. In terms of time since the first running away, the contrast between adolescents who had initiated running away within the previous 6 months and those who had started more than 6 months ago was less prominent, with older age, having a job, and sexual experience showing increased prevalence in the latter group.

**Table 1:** Sample characteristics of the adolescents age 12--18 for running away in the 2004--2006 street outreach programme in Taiwan.

		Severity		Time since the first running away	
			
	Never	Trial^b^	Extended^c^	< 6	≥ 6
	ran away	running away	running away	months	months
	(n = 15507)	(n = 441)	(n = 1141)	(n = 176)	(n = 1450)
Variables^a^	n (%)	n (%)	n (%)	n (%)	n (%)
Male gender	7258 (46.8)	229 (51.9)*	654 (57.3)*	87 (49.4)	821 (56.6)*
Age 16--18 (vs. 12--15)	9286 (59.9)	257 (58.3)	742 (65.0)* ^§^	75 (42.6)*	945 (65.2)* ^§^
Single-parent family	1872 (12.1)	93 (21.1)*	355 (31.1)* ^§^	54 (30.7)*	405 (28.0)*
Not attending school	326 (2.1)	21 (4.8)*	142 (12.5)* ^§^	18 (10.2)*	148 (10.2)*
Having a job	1800 (11.6)	102 (23.2)*	395 (34.6)* ^§^	42 (23.9)*	462 (31.9)* ^§^
Weekly allowance (NTD)^d^					
0--500	7562 (48.8)	196 (44.4)	488 (42.8)*	77 (43.8)	627 (43.2)*
501--1500	6321 (40.8)	193 (43.8)	464 (40.7)*	74 (42.1)	603 (41.6)*
≥ 1501	1622 (10.5)	52 (11.8)	189 (16.6)*	25 (14.2)	220 (15.2)*
Truancy	4054 (26.1)	277 (62.8)*	913 (80.0)* ^§^	139 (79.0)*	1080 (74.5)*
Sexual experience	1131 (7.3)	88 (20.0)*	434 (38.0)* ^§^	45 (25.6)*	483 (33.3)* ^§^

^a^Some variables have missing values: 6 for single-parent family, 1 for having a job, and 2 for weekly allowance

^b^This refers to running away only 1 time and lasting 1 day or less that did not occur within the past 6 months (44 subjects were deleted from this analysis because of a runaway occurrence within the past 6 months).

^c^This refers to running away at least 2 times or having a longest runaway time of > 1 day

^d^New Taiwan Dollars (1 USD ≅ 30 NTD)

*p value < 0.05 for χ^2 ^test in comparison with never running away

^§^*p *value < 0.05 for χ^2 ^test in comparison with trial running away (by severity) or within 6 months since onset (by time-since-onset)

### Running away and substance use

The relationship between running away and substance use is displayed in Table 2. First, when lifetime use of tobacco, alcohol, betel nut, and illegal drugs/inhalants are compared among adolescents grouped by the severity of running away, the prevalence of drug use increased from those who had never ran away to those with a trial running away, to those with an extended running away. In the multivariable logistic regression analysis with adjustment for regional clustering design effects, survey year, and potential confounders (i.e., the covariates reported in Table 1), running away remained significantly associated with a higher risk of lifetime use of alcohol, tobacco, betel nut, and illegal drugs/inhalants, with adjusted ORs (aORs) ranging from 1.4 (95% confidence interval [CI]: 1.0-2.1) to 1.9 (95% CI: 1.5-2.4) for adolescents with a trial running away and from 1.9 (95% CI: 1.7-2.2) to 3.0 (95% CI: 2.3-3.9) for those with an extended running away. When adolescents with extended running away were compared to those with a trial running away, the former had a greater risk across all substance usage, with aORs (95% CI) being 1.4 (1.1-1.8), 1.6 (1.3-2.1), 1.9 (1.3-3.0), and 2.1 (1.3-3.3) for alcohol, tobacco, betel nut, and illegal drug/inhalant use, respectively (data not shown in Table 2).

**Table 2:** Logistic regression analysis of substance use on running away among the adolescents of the 2004--2006 street outreach programme in Taiwan.

		Alcohol use		Tobacco use	
			
Experience of running away	N	n (%)	aOR^a ^(95% CI)	n (%)	aOR^a ^(95% CI)
**Lifetime use model**					
Severity of running away					
Never ran away	15507	4520 (29.2)	1.0	2232 (14.4)	1.0
Trial running away^b^	441	217 (49.2)	1.5 (1.2-1.8)	164 (37.2)	1.9 (1.5-2.4)
Extended running away	1141	724 (63.5)	1.9 (1.7-2.2)	657 (57.6)	2.9 (2.5-3.4)
**Past 6-months' use model**					
Time since the first running away					
Never ran away	15507	3809 (24.6)	1.0	1523 (9.8)	1.0
< 6 months	176	94 (53.4)	2.0 (1.4-2.8)	68 (38.6)	2.6 (1.7-3.8)
≥ 6 months	1450	773 (53.3)	1.7 (1.5-2.0)	603 (41.6)	2.5 (2.1-2.8)

		**Betel nut use**		**Illegal drug/inhalant use**	
			
**Experience of running away**	**N**	**n (%)**	**aOR^a ^(95% CI)**	**n (%)**	**aOR^a ^(95% CI)**

**Lifetime use model**					
Severity of running away					
Never ran away	15507	400 (2.6)	1.0	239 (1.5)	1.0
Trial running away^b^	441	29 (6.6)	1.4 (1.0-2.1)	26 (5.9)	1.6 (1.0-2.6)
Extended running away	1141	173 (15.2)	2.4 (1.9-3.1)	190 (16.7)	3.0 (2.3-3.9)
**Past 6-months' use model**					
Time since the first running away					
Never ran away	15507	235 (1.5)	1.0	171 (1.1)	1.0
< 6 months	176	7 (4.0)	1.1 (0.5-2.6)	13 (7.4)	2.1 (1.1-4.3)
≥ 6 months	1450	142 (9.8)	2.5 (1.9-3.3)	147 (10.1)	2.6 (1.9-3.4)

^a^Estimates obtained from a logistic regression model, which took region clustering design effects into account and adjusted for survey year, gender, age, family structure, attending school, having a job, weekly allowance, truancy, and sexual experience

^b^With 44 subjects deleted from this analysis because they had a single episode of running away that lasting 1 day or less and was within the past 6 months

Next, recent substance use was compared among adolescents grouped by the recentness of the first running away (lower part of Table 2). Compared with those who had never absconded, the risk of recent use of a substance was increased for both recentness < 6 months and recentness ≥ 6 months except for betel nut, which was increased only for recentness ≥ 6 months. If adolescents with recentness ≥ 6 months are compared with those with recentness < 6 months, the former had a greater risk only for recent betel nut use, with aORs (95% CI) of 0.9 (0.6-1.2), 1.1 (0.7-1.5), 2.5 (1.1-5.6), and 1.3 (0.5-2.7) for alcohol, tobacco, betel nut, and illegal drug use, respectively (data not shown in Table 2). It needs to be noted that, despite the magnitude of the OR for illegal drug/inhalant use, this failed to reach statistical significance probably due to the small number of subjects with such an experience within the last 6 months.

### Initiation of running away vs. initiation of substance use

When substance was grouped into four major categories (i.e., alcohol, tobacco, betel nut, and illegal drug/inhalant), 16% to 19% of adolescents reported that both the first absconding event and substance use occurred at the same age (Table 3). Alcohol and tobacco use was more frequently initiated before the absconding event (42.6% and 45.8%, respectively) than after absconding (38.9% and 35.1%, respectively). Betel nut use was initiated more frequently after the first absconding event (48.0%) than before absconding (33.3%). Among the illegal drug/inhalant use reported by those with running away, ecstasy was most common drug used, followed by ketamine, marijuana, methamphetamine, flunitrazepam, and glue. For illegal drug/inhalant use as well as the composite variables (i.e., any one of them), the absconding event preceded the initiation of drug use for the majority of adolescents (> 60%). The results of a chi-square analysis on the ordering between running away and substance use showed that the initiation of substance use statistically significant for after the first absconding event for all substances (*p *< 0.05) except for alcohol.

**Table 3:** Age at onset of substance use compared with age at which the subject first ran away among the adolescents of the 2004--2006 street outreach programme in Taiwan

Substance	N	Younger	Same Age	Older	*p *value for
		n (%)	n (%)	n (%)	χ ^2 ^test^a^
Alcohol	937	399 (42.6)	173 (18.5)	365 (38.9)	0.2187
Tobacco	813	372 (45.8)	156 (19.2)	285 (35.1)	0.0007
Betel nut	198	66 (33.3)	37 (18.7)	95 (48.0)	0.0223
Any illegal drug/inhalant	211	40 (19.0)	31 (15.7)	140 (66.3)	<0.0001
Ecstasy	139	25 (18.0)	21 (15.1)	93 (66.9)	<0.0001
Ketamine	75	13 (17.3)	9 (12.0)	53 (70.7)	<0.0001
Marijuana	33	5 (15.2)	7 (21.2)	21 (63.6)	0.0017
Methamphetamine	25	5 (20.0)	2 (8.0)	18 (72.0)	0.0067
Flunitrazepam	26	6 (23.1)	3 (11.5)	17 (65.4)	0.0218
Glue	26	2 (7.7)	8 (30.8)	16 (61.5)	0.0010

^a^The proportions of those with a younger or older age at onset for substance use were tested against the expected proportions of 50% under the null hypothesis of no ordering between running away and substance use.

### Reasons of the first running away and substance use

In the 2006 survey, it was found that 76.4% (n = 356) of adolescents first ran away due to dissatisfaction with their home life, while 19.1% (n = 89) said they ran away for the excitement of it, and 4.5% (n = 21) said it was for both reasons (Table 4). Compared to adolescents who had never ran away, those who had absconded, regardless of the reason, had a higher risk of substance use. When those who were running from unsatisfactory home life were compared to those who absconded for excitement, their risks for alcohol use were similar but the former tended to have a higher risk of tobacco, betel nut, and illegal drug/inhalant use. However, the differences between the two groups did not reach statistical significance, with the *post hoc *pairwise comparisons in aORs (95% CI) being 1.1 (0.7-1.7), 1.3 (0.7-2.2), 1.8 (0.8-3.9), and 1.5 (0.7-3.4) for alcohol, tobacco, betel nut, and illegal drug use, respectively.

**Table 4:** Logistic regression analysis of lifetime substance use on the reason of the first running away among the adolescents of the 2006 street outreach programme (n = 5695^a^) in Taiwan.

		Alcohol use		Tobacco use	
Reason of the first running away	N	n (%)	aOR^b ^(95% CI)	n (%)	aOR^b ^(95% CI)
Never ran away	5229	1256 (24.0)	1.0	666 (12.7)	1.0
Running from unsatisfactory family life	356	202 (56.7)	1.9 (1.5-2.4)	194 (54.5)	3.1 (2.4-4.0)
Running to excitement	89	52 (58.4)	1.9 (1.2-2.9)	48 (53.9)	2.5 (1.5-4.1)
Both	21	11 (52.4)	2.2 (0.9-5.3)	12 (57.1)	4.1 (1.5-11.6)

		**Betel nut use**		**Illegal drug/inhalant use**	
**Reason of the first running away**	**N**	**n (%)**	**aOR^b ^(95% CI)**	**n (%)**	**aOR^b ^(95% CI)**

Never ran away	5229	119 (2.3)	1.0	59 (1.1)	1.0
Running from unsatisfactory family life	356	55 (15.5)	3.1 (2.1-4.7)	47 (13.2)	3.5 (2.2-5.5)
Running to excitement	89	10 (11.2)	1.7 (0.8-3.6)	9 (10.1)	2.5 (1.1-5.4)
Both	21	1 (4.8)	0.8 (0.1-5.8)	2 (9.5)	2.4 (0.5-12.5)

^a^52 subjects with missing information on the reason for the first running away were not included in this analysis.

^b^Estimates obtained from a logistic regression model that took region clustering design effects into account and adjusted for survey year, gender, age, family structure, attending school, having a job, weekly allowance, truancy, and sexual experience.

## Discussion

Our results among adolescents who were still living with their family reveal that the risk for lifetime use of the four kinds of psychoactive substances increased steadily from adolescents with a trial absconding event to those with extended running away when compared to those without such experience. Furthermore, a longer elapsed time since the first absconding episode was associated with a greater risk of betel nut or illegal drug/inhalant use in the past 6 months, but not tobacco or alcohol use. Similar differential associations were also found for the relationship between initiation age of substance use and the first absconding event as well as between substance use and reason for running away. These results help shed light on the relationships between running away from home and substance use, and have implications for intervention and prevention of adolescent substance use.

This study has a distinguished feature in that it adopted an outreach approach to recruit adolescents who would otherwise not be captured in school- or household- based surveys [26,29]. We focused on adolescents interviewed on the streets during regular school days and weekends, regardless of their living or school-attending situation. In addition, our samples were drawn from cities/towns nationwide in an effort to capture regional differences. In our 3-year outreach sample, around 10% of the participants reported an absconding experience, with about 30% of them being trial and the remaining 70% being extended; overall, about 10% had occurred within 6 months since onset and 90% had occurred beyond 6 months since onset. Thus, at least to some extent, our outreach approach did succeed in recruiting adolescents whose absconding covered a broad spectrum.

Running away severity-associated increased risk in lifetime substance use appears across all types of psychoactive substance; even for adolescents with trial running away, whose increased risk of substance use was salient to illegal drug/inhalant use. Extending current understanding on substance use among homeless youth [1,2,11,16], the higher prevalence of drug use among adolescents with trial running away could be explained by their dysfunctional families or their experience of physical, emotional, or sexual abuse, since the majority considered an unsatisfactory family life as the primary cause for running away (~ 81%) and a higher proportion came from single-parent families (21% vs. 12%) compared to those without any absconding experience. Previous studies have also indicated that adolescents with an unsatisfactory family life may use substances as a way to escape from their suffering [32,33]. Nevertheless, even after adjustment for these adverse sociodemographic and behavioural characteristics, trial running away was still associated with substance use. This indicates that experience as a runaway *per se *increases the risk of adolescent involvement with psychoactive substances.

In contrast, the recentness of the first absconding experience exhibited a different pattern in relation to substance use in the past 6 months. The increased risk of recent use of both alcohol and tobacco did not differ between adolescents with a recentness of < 6 months and those with a recentness of ≥ 6 months. In addition, the risk of recent use of betel nut and possibly of illegal drug/inhalant increased for those with a recentness of ≥ 6 months compared with that for those with a recentness of < 6 months. This indicates that running away has a long-term influence on betel nut and illegal drug use. In light of the independence between severity in absconding and recentness of the first running away, our results highlight the importance of the latter in predicting a subsequent progression toward more advanced substance use.Similarly, the comparisons of initiation age for absconding with that of the four substance categories further supports the hypothesis that tobacco and alcohol involvement among adolescents who are in an early stage of running away is more likely to be pre-existing [13], whereas their use of betel nut or illegal drugs or inhalants may mainly be a result of the running away itself. This may be because tobacco and alcohol are relatively more available (can be purchased from convenience stores) than betel nut (usually sold in stalls along roadside in suburban districts), illegal drugs (not available in public), and inhalants (not used in public). Consequently, betel nut and illegal drugs/inhalants may be less accessible to adolescents until they abscond. Previous studies in Taiwan also found that the average age of starting betel nut use is older than for tobacco use among adolescents [24]. Thus, taken together, our findings indicate that adolescents at an early stage within the running away spectrum are already in a progression towards more advanced substance use.

When the reasons for running away from home were compared, adolescents who were running from an unsatisfactory family life appeared to have a slightly higher risk of using tobacco, betel nut, and illegal drugs than those who absconded for excitement. Although the difference did not reach statistical significance (probably as a result of small size of the latter group and the fact they were in the early stage of the running away), it echoes the finding of a previous study conducted on shelter or street youths [19]. More importantly, our results indicate that once adolescents leave home, they develop additional psychoactive substance problems, regardless of the reasons for their running away [34-36].

Our findings have implications for prevention or intervention of adolescent substance use by parents/primary caregivers, teachers, and healthcare workers. The initiation of running away is a critical time for such adolescents because they may engage in substance use during this period [37]. Since adolescents at an early stage of running away usually return home after a short period of time, their running away can be detected easily by the caregivers. For this reason, care givers are pivotal in helping to terminate the adolescent absconding [38]. In particular, if adolescents have an unsatisfactory family life, they may run away again and use substances as a way to escape from their suffering. In addition, teachers and health care workers, who encounter children or adolescents who are experiencing stressful household environments, can play important roles as family health advisors in order to reduce the adolescent's use of psychoactive substances [39]. Furthermore, an adolescent service centre can provide runaway adolescents with a channel for seeking help or obtaining consultations and this can try to bring about a reunification of the runaway adolescents with their families [40].

Our results need to be interpreted with some limitations in mind. First, our use of a paper-and-pencil questionnaire for the measurement of sensitive behaviours could result in underreporting of drug use. However, such underreporting was unlikely to be associated with the adolescents' experience of running away and, thus, misclassification would make little, if any, difference to the results. This might lead to underestimation rather than overestimation of the odds ratios linking running away to substance use. Second, the cross-sectional nature of this study is unable to clearly distinguish a causative pathway between running away and substance use; a future longitudinal study is warranted to confirm our findings. Third, the exact members in each family living with a respondent were not queried in the questionnaire. Since some adolescents might live with grandparents only or with both their parents and grandparents in Taiwan, the lack of such information render us unable to evaluate whether parents exert a differential effect in terms of supervision over an adolescent's behaviour than grandparents. Fourth, since our participants were selected in a non-probabilistic manner, some selection bias might have occurred and the sample might not reflect the lifestyle of all adolescents wandering on the streets.

## Conclusion

This study among adolescents who were recruited via outreach and still living with their family indicated that more significant running away and a longer time since the first absconding experience were associated with more advanced substance involvement. Comparing the temporal sequence linking substance use and the first absconding episode, both alcohol and tobacco use were most frequently initiated before the first running away, whereas both betel nut and illegal drug/inhalant use were most frequently initiated after this event. Once adolescents had left home, they developed additional psychoactive substance problems, regardless of their reasons for running away. These findings have implications for caregivers, teachers, and healthcare workers when trying to prevent and/or intervening in adolescent substance use.

## Competing interests

The authors declare that they have no competing interests.

## Authors' contributions

S-HW was responsible for data analyses and led the writing of the article. W-CC supervised the fieldwork of the outreach program and assisted data management and analysis. C-YL-T and C-YC assisted with the analyses and writing. WJC was the principal investigator, contributed to the conceptualization of the original research, and oversaw all aspects of its implementation. All authors helped to conceptualize ideas, interpret findings, and edit drafts. All authors read and approved the final manuscript.

## Pre-publication history

The pre-publication history for this paper can be accessed here:

http://www.biomedcentral.com/1471-2458/10/29/prepub
